# Acceptability, Relevance, and Short-Term Outcomes of the STAC-T Bullying Bystander App: Feasibility Quantitative Study

**DOI:** 10.2196/76830

**Published:** 2025-11-18

**Authors:** Diana M Doumas, Aida Midgett, Robin Hausheer, Amanda Winburn, Mary K Buller, Taylor Perron, Jennalyn Shelton, Brandon Herbeck

**Affiliations:** 1 Department of Counselor Education, Boise State University, 1910 University Drive, Boise, ID, 83725, United States, 1 2084261219; 2 Institute for the Study of Behavioral Health and Addiction, Boise State University, Boise, ID, United States; 3 Department of Counseling, Human Development and Family Science, University of Vermont, Burlington, VT, United States; 4 Department of Leadership and Counselor Education, University of Mississippi, Oxford, MS, United States; 5 Klein Buendel Inc., Golden, CO, United States

**Keywords:** technology-based bullying intervention, bullying, acceptability, relevance, middle school

## Abstract

**Background:**

Bullying is a significant public health issue, with approximately 25% of middle school students reporting being a target of bullying in the past year. Students who are targets of bullying or witness bullying are at high risk for negative mental health outcomes, including depression and anxiety. STAC is an evidence-based bullying bystander intervention for middle school students, with program outcomes that include reductions in bullying perpetration and victimization, as well as associated mental health risks. We developed a technology-based version of STAC (STAC-T) to reduce implementation barriers associated with in-person bullying prevention programs. STAC-T is an interactive app that includes a 40-minute training and a 15-minute booster session.

**Objective:**

This study aimed to evaluate the acceptability and relevance of the STAC-T program. We were also interested in how program acceptability and relevance are related to the use of specific bystander intervention skills (eg, STAC strategies) students learn in the program.

**Methods:**

This study was part of a larger study in which students recruited from 6 middle schools in rural, low-income communities in the United States were randomized to either the STAC-T intervention or a control condition. Participants in this study were 229 students in the intervention group who completed the 30-day follow-up survey, including the acceptability and relevance questionnaire. The survey assessed program acceptability and relevance, whether or not students witnessed bullying posttraining, and the use of the STAC strategies to intervene in bullying situations. Descriptive statistics were used to assess acceptability, relevance, and the use of STAC strategies. Linear regression analysis was used to assess the relationship of program acceptability and relevance to STAC strategy use.

**Results:**

Of the 229 student participants, the majority reported the program was acceptable (188, 82.1%, to 206, 90.0%) and relevant (180, 78.6%, to 190, 83.0%) for students at their school. Of the 54.6% (125/229) of students who witnessed bullying posttraining, 88.8% (111/125) reported the use of at least one STAC strategy to intervene when witnessing bullying. Students were most likely to use the STAC bystander intervention strategies *Turning it Over* and *Accompanying Others*, relative to *Stealing the Show* and *Coaching Compassion*. Regression analyses revealed that program relevance was a significant predictor of posttraining use of STAC strategies (*P*=.016). In contrast, program acceptability was not a significant predictor of posttraining STAC strategy use (*P*=.660).

**Conclusions:**

This study provides support for the acceptability and relevance of STAC-T and its effectiveness in promoting the use of the STAC strategies to intervene in bullying situations. Furthermore, program relevance was related to STAC strategy use, highlighting the importance of assessing program relevance for specific student populations.

## Introduction

### Background

Bullying is a serious problem among youth in the United States. Bullying has been described as an often-repeated, unwanted, intentional aggressive behavior that takes place within the context of a relationship with a perceived power imbalance [1,2]. Bullying can occur in the forms of physical (eg, hitting, kicking, and damaging personal property), verbal (eg, name calling and threats), relational (eg, spreading rumors and social exclusion), and online (eg, cyberbullying) behavior [3]. Bullying peaks in middle school, with 26.3% of middle school students reporting bullying victimization in the past year [4]. Comprehensive, school-wide bullying programs are effective in reducing bullying [5]; however, comprehensive, school-wide programs require significant resources, creating barriers to implementation [6], particularly for schools in rural and low-income communities [7]. Technology-based interventions provide a promising platform for bullying bystander prevention programming, as these programs can be easily disseminated to large groups of students at little cost relative to in-person, comprehensive, school-wide bullying prevention programs. Thus, technology-based interventions have the potential to improve access to programming and decrease implementation barriers, particularly in rural communities [7].

### Bystander Training

Students who witness bullying as bystanders can engage in bullying in several ways, including taking on the following roles: (1) *assistants* who join in the bullying behavior, directly helping the student who is bullying; (2) *reinforcers* who encourage the student who is bullying by laughing or cheering; (3) *outsiders* who ignore or walk away from bullying situations; and (4) *defenders* who attempt to intervene in the bullying situation [8]. Research suggests that approximately 80% of students witness bullying as bystanders [9], yet only 20% intervene in bullying situations [10]. Students may not intervene because they do not know what to do when witnessing bullying [11] or they may intervene in maladaptive ways (eg, aggressive behavior) [12] and become socially rejected by peers [8]. Thus, bystander training is essential for students’ acquisition of prosocial skills they can use to intervene in bullying situations. However, many comprehensive, school-wide programs do not include bystander training [5].

### The STAC Bullying Bystander Intervention

STAC [13] is a brief, in-person bullying bystander intervention developed to teach students who witness bullying how to intervene using prosocial skills. The training is 75 minutes and includes education about bullying, consequences associated with bullying, the 4 bystander roles, and the 4 STAC strategies: (1) *Stealing the Show*, using humor or distraction to interrupt the bullying situation; (2) *Turning it Over*, informing an adult about the bullying incident; (3) *Accompanying Others*, providing support to the targeted student; and (4) *Coaching Compassion*, gently confronting the student who bullied to increase empathy for the target. The training also includes an experiential component that includes role-plays during which students are presented with bullying scenarios and then practice using the STAC strategies to intervene in the bullying situation. The STAC training is followed by two 15-minute booster sessions to reinforce skill acquisition learning. Research indicates the STAC intervention is effective in decreasing bullying victimization [14-16] and perpetration [15,17,18] and reducing mental health risks [17-23] among students trained in the program.

### The STAC-T App

The STAC-T app is a technology-based version of the in-person STAC bullying bystander intervention. The STAC-T app was developed to address specific needs and barriers identified by school personnel in rural, low-income communities [24]. Prototype development included focus groups and interviews with school personnel and students followed by usability testing [25]. The resulting fully built-out STAC-T app is a 40-minute training followed by one 15-minute booster session. Initial research on the fully built-out STAC-T app indicates that the STAC-T app has high usability, acceptability, and relevance among key stakeholders in rural, low-income communities [26]. Specifically, 10 school personnel and 11 middle school students in 2 states completed usability testing and individual interviews. Quantitative results indicated that both school personnel and students found the program to be highly usable (eg, easy to use, acceptable, and feasible) and reported high program satisfaction. Qualitative findings supported these results, with both school personnel and students providing feedback that the STAC-T app was useful, relevant, and appropriate.

### This Study

When developing intervention programs, it is important to establish acceptability (eg, how the user perceives the program) and program relevance (eg, social validity), as acceptability is related to successful program outcomes [27] and perceived relevance by the end user is associated with the use of intervention skills [28]. Although research conducted in the STAC-T development provides initial support for program usability, program acceptability and relevance were assessed using a small sample (n=21) and were not assessed in relation to use of skills posttraining. Thus, the purpose of this study was to evaluate the acceptability and relevance of the STAC-T app, as well as posttraining use of the STAC strategies, among a large, more diverse sample of middle school students. To achieve this aim, we assessed program acceptability, relevance, and STAC strategy use among students who completed the 30-day follow-up assessment and who were randomly assigned to the intervention group in a larger efficacy trial (n=249). Acceptability, relevance, and strategy use were evaluated after completion of both STAC-T training modules and the booster session. Furthermore, as previous research suggests, acceptability is positively related to program outcomes [27] and relevance is positively related to the use of intervention skills [28], we were also interested in how program acceptability and relevance were related to the use of the STAC strategies posttraining. The study had the following objectives: (1) to assess acceptability and relevance of the STAC-T app, (2) to assess STAC strategy use posttraining, and (3) to evaluate the relationship of acceptability and relevance to use of the STAC strategies.

## Methods

### Participants

Participants were recruited as part of a larger study (Clinicaltrials.gov; NCT05572398 [29]) conducted at 6 middle schools in rural, low-income communities in Northwest, Southern, and Eastern regions of the United States. Parents of all students in sixth through eighth grade (N=1802) were sent consent forms. A total of 40.84% (736/1802) of students provided consent. Of the 736 students, 34.0% (n=613) agreed to participate in the study, and 51.1% (n=307) were assigned to the intervention condition. Among students in the intervention condition, 81.1% (249/307) completed the 30-day follow-up survey and were included in this study. Participant age ranged from 11 to 15 (mean 12.1, SD 0.9) years, with 39.8% (99/249) in grade 6, 43.0% (107/249) in grade 7, and 17.3% (43/249) in grade 8. Students self-reported gender as female (136/249, 54.6%) and male (100/249, 40.2%), with 5.2% (13/249) missing data. Students self-reported ethnicity/racial background as White (108/249, 43.4%), Hispanic or Latino (60/249, 24.1%), Black or African American (46/249, 18.5%), more than one race (21/249, 8.4%), Asian American (3/249, 1.2%), American Indian or Alaska Native (3/249, 1.2%), Native Hawaiian or Other Pacific Islander (1/249, 0.4%), and other (4/249, 1.6%), with 1.2% (3/249) missing data.

### Procedures

Participant recruitment occurred during the fall of 2024. Recruitment procedures were conducted through a common core course for each of the 3 grade levels. All parents of sixth- through eighth-grade students received a prenotification email from the school principal informing them of the study. Moreover, research team members hand-delivered a cover letter explaining the study and a copy of the informed consent to students. Students were asked to provide their parents with the documents and to return informed consent documents to their teacher. The school also sent a notification email to parents with a cover letter and informed consent form with a link for parents to sign providing consent electronically. Teachers reminded students to return the informed consent forms. The school also sent parents a reminder email with a link to sign the informed consent form electronically from the principal. For students whose parents consented, a research team member conducted assent immediately before baseline data collection.

Class periods were randomly assigned to the STAC-T intervention or assessment-only control condition using block randomization [30]. Block randomization ensures relatively equivalent group sizes by dividing participants into blocks and randomly assigning condition within each block. In this study, the blocks were the number of periods at the school. Block randomization was conducted for each school as the number of periods varied by school, ranging from 2 to 8 periods. Across the 6 schools, there were 29 periods, with 15 periods randomized to the control condition and 14 periods randomized to the intervention condition. A member of the research team went into each participating classroom and described the research study and invited the students with parental consent to participate. Students were given a unique personal identification number (PIN) to maintain confidentiality and a URL to access the study website where they read a welcome screen explaining the research and were asked for their assent to participate. Once students gave assent by clicking “agree,” they were taken to a screen that asked them to enter their PIN and were then directed to begin the baseline survey, which took between 20 and 30 minutes. Students in the intervention condition completed the STAC-T intervention and booster session over the next 3 weeks. Students without parental consent and those who did not provide consent completed an alternate activity in the classroom. Students in the intervention condition who completed the baseline survey were invited to participate in the 30-day follow-up survey used in this study.

### Ethical Considerations

All study procedures were approved by the Boise State University Institutional Review Board (101-SB21-205) and by the School Districts or Administration. We used active informed consent for this study. Parents were provided with an informed consent form that contained information about the study, including purpose and background, procedures, risks or discomforts, extent of confidentiality (eg, students provided a unique study PIN and study data deidentified), benefits, payment, and how to contact the primary investigator and the Boise State University Institutional Review Board. Students with parental consent were provided with an informed assent form that contained the same information. Incentives included a US $100 gift card for teachers and donuts, oranges, and stickers for students after completion of the follow-up survey.

### The STAC-T App

**Figure 1.**
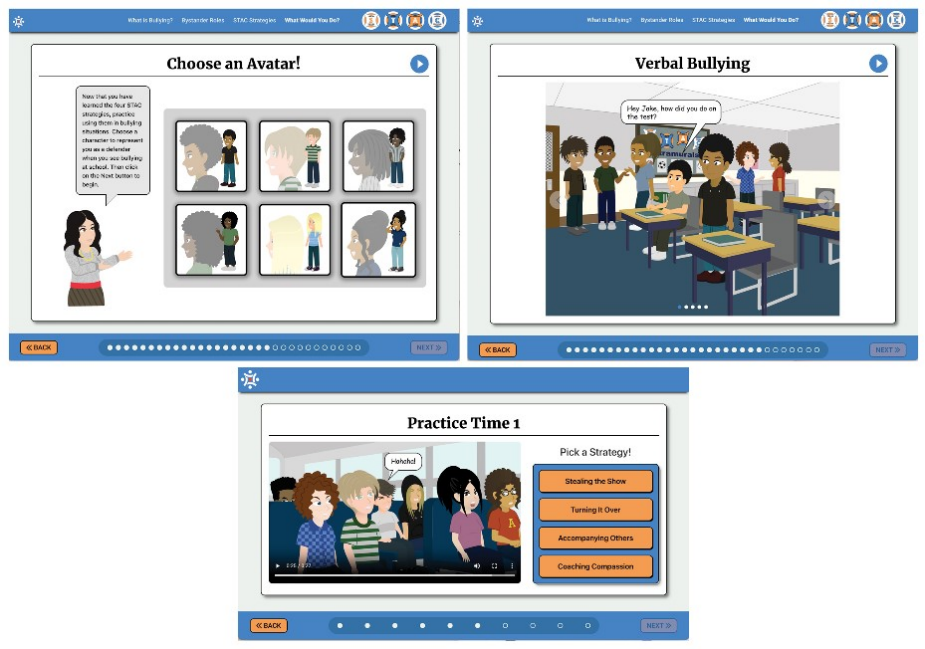
Samples from the STAC-T app.

The STAC-T app (Figure 1) is a 40-minute training followed by one 15-minute booster session. The educational modules include the following content: (1) What is bullying?—users are presented with background information on bullying, including definitions of bullying, bullying facts and statistics, characteristics of students who bully, and negative consequences associated with bullying; (2) What are bystanders?—this module explains the 4 bystander roles: (a) *assistants*: those who directly join in to help the bully, (b) *reinforcers*: those who encourage the bully, (c) *outsiders*: those who do nothing or walk away, and (d) *defenders*: those who do something to intervene; and (3) STAC strategies: users were introduced to the 4 STAC strategies: (a) *Stealing the Show*: using humor or distraction to interrupt the bullying situation, (b) *Turning it Over*: informing an adult about the bullying incident, (c) *Accompanying Others*: providing support to the targeted student, and (d) *Coaching Compassion*: gently confronting the student who bullied to increase empathy for target. Users are then guided through STAC strategy practice in which students select an avatar, view bullying scenarios, select each STAC strategy and view the avatar enacting the selected strategy, and receive feedback on strategy effectiveness. An artist hand-illustrated and styled 6 avatars with light-, medium-, and dark-colored hair in different styles, as well as light, medium, and dark skin tones for students to choose from to best represent themselves. The booster session included additional practice with bullying scenarios and STAC strategy use. “Badges” (visual reward icons; eg, *Stealing the Show Badge*) are included throughout the app to reward learning and encourage user engagement and adherence.

### Measures

#### Acceptability and Relevance of the STAC-T App

Acceptability (ie, ease of use and utility) and relevance of the STAC-T app were assessed using a social validity survey that comprised 8 items [31]. Items were ranked on a 4-point Likert scale, ranging from 1 (*strongly disagree*) to 4 (*strongly agree*). Items were subjected to a principal component factor analysis to confirm the factors underlying the social validity survey. An oblique rotation was used to allow for correlation among the items. Eigenvalues showed that the first component (ie, acceptability) explained 69.0% of the variance, the second component (ie, relevance) explained 9.9% of the variance, and the remaining components had eigenvalues <1. A 2-factor solution, which explained 78.9% of the variance, was selected. Acceptability items were as follows: “The STAC-T training was easy to understand,” “The STAC-T program was useful,” “The STAC-T training was interesting,” “I learned something from the STAC-T program,” and “I would recommend the STAC-T program to other students at my school” (α=.92). Relevance items were as follows: “The STAC-T training information was relevant for students at my school,” “The STAC-T training examples of bullying were relevant for schools like my school,” and “The STAC-T strategy role-plays were relevant for students at my school” (α=.89).

#### Use of STAC Strategies

The use of each STAC strategy was measured by a single item. Students were asked, “How often would you say that you used these strategies to stop bullying in the past month? (a) Stealing the Show – using humor to get the attention away from the bullying situation, (b) Turning it Over – telling an adult about what you saw, (c) Accompanying Others – reaching out to the student who was the target of bullying, and (d) Coaching Compassion – helping the student who bullied develop empathy for the target.” Items were rated on a 5-point Likert scale, ranging from 1 (*never/almost never*) to 5 (*always*/*almost always*). The items were summed to create a total scale (α=.81). Prior research has used these items to examine the use of STAC strategies posttraining with middle school students [17,32,33].

#### Bystander Status

We assessed bystander status with the following question: “Have you seen bullying at school in the past month?,” with response choices of *Yes* and *No*. Researchers have used this item to measure bystander status among middle school students [16,17,34]. Using this measure, 54.6% (125/229) of students were classified as bystanders.

### Data Analysis

Data from the questionnaires were analyzed using the Statistical Package for the Social Sciences (version 25.0). Before conducting statistical analyses, the data were examined for outliers and normality, and all variables were within the normal range for skew and kurtosis. Of the 249 students who completed the follow-up survey, 92.0% (n=229) completed the acceptability and relevance questionnaire. The 8.0% (n=20) who did not complete the acceptance and relevance questionnaire were omitted from the data analyses. Missing data for the remaining participants were imputed using expectation-maximization. Missingness was <.05% for acceptability and relevance, <2% for use of STAC strategies, and 0% for bystander status. To evaluate intervention acceptability, relevance, and use of STAC strategies, frequency data were combined into dichotomous variables. For acceptability and relevance, “strongly disagree” and “disagree” were combined to form a disagree category, and “strongly agree” and “agree” were combined to create an agree category. For use of STAC strategies, responses were categorized as not used (score of 0) and used (score >0). To examine the relationship between intervention acceptability and relevance and use of STAC strategies, we conducted a linear multiple regression analysis using the original frequency data for acceptability, relevance, and total use of STAC strategies. We entered acceptability and relevance into the model simultaneously. We examined the variance inflation factor (VIF) for the predictors to assess multicollinearity. We calculated effect size using *R*^2^ for the regression analysis, with magnitude of effects interpreted as follows: small=0.01, medium=0.09, and large=0.25 [35]. All analyses were considered significant at *P=*.04.

## Results

### Acceptability and Relevance

Means, SDs, and percent agreement for acceptability and relevance items are reported in Table 1. As seen in Table 1, the majority of students reported the program was acceptable (188/229, 82.1%, to 206/229, 90.0%) and relevant (180/229, 78.6%, to 190/229, 83.0%) for students at their school. All acceptability and relevance items were rated >3.00 on a 1- to 4-point scale.

**Table 1.** Participants reporting agreement with acceptability and relevance (N=229).

Item^a^	Values, mean (SD)	Agreement, n (%)
Acceptability (mean 3.22, SD 0.69)		
The STAC-T training was easy to understand	3.23 (0.75)	206 (90.0)
The STAC-T training was useful	3.20 (0.79)	196 (85.6)
The STAC-T training was interesting	3.18 (0.82)	193 (84.7)
I learned something from the STAC-T program	3.31 (0.80)	203 (88.6)
I would recommend the STAC-T program to other students at my school	3.22 (0.85)	188 (82.1)
Relevance (mean 3.07, SD 0.75)		
The STAC-T training information was relevant for students at my school	3.13 (0.80)	190 (83.0)
The STAC-T training examples of bullying were relevant for students at my school	3.09 (0.84)	190 (83.0)
The STAC-T strategy role-plays were relevant for students at my school	3.21 (0.83)	180 (78.6)

a Responses were scored on a 4-point Likert scale, ranging from 1=strongly disagree to 4=strongly agree.

### Use of the STAC Strategies

Results for student self-reported use of each STAC strategy are presented in Table 2. Results are reported for students who reported witnessing bullying in the past 30 days (125/229, 54.6%). As seen in Table 2, the majority of students (111/125, 88.8%) used at least one STAC strategy posttraining, with higher rates reported for the strategies *Turning it Over* and *Accompanying Others* relative to *Stealing the Show* and *Coaching Compassion*.

**Table 2.** Number of students reporting use of strategies (n=125)*.*

Strategies	Used strategy, n (%)	Did not use strategy, n (%)
Stealing the Show	73 (58.4)	52 (41.6)
Turning it Over	86 (68.8)	39 (31.2)
Accompanying Others	97 (77.6)	28 (22.4)
Coaching Compassion	73 (58.4)	52 (41.6)
Total strategies^a^	111 (88.8)	14 (11.2)

a Total strategies=used at least one STAC strategy.

### The Relationship Between Acceptability, Relevance, and Use of STAC Strategies

We conducted a linear multiple regression analysis to examine the unique effect of acceptability and relevance on use of STAC strategies among students who reported witnessing bullying posttraining. Before conducting the regression analyses, we examined bivariate correlations for predictor and outcome variables (Table 3).

**Table 3.** Bivariate correlations for acceptability, relevance, and use of STAC strategies.

Measure	1	2	3
Acceptability	—^a^	—	—
Relevance	0.77^b^	—	—
Use of strategies	0.20^c^	0.29^b^	—

a Not applicable.

b *P*<.001.

c *P*=.031.

Although acceptability and relevance were highly correlated, the observed VIF was 2.4, with corresponding tolerance at 0.4, which is below the rule of thumb of VIF <10 [36], suggesting acceptable levels of multicollinearity among the predictor variables. The full regression equation was significant (*R*^2^=0.09; *F*_112, 2_=5.38; *P*=.006). Relevance was a significant predictor of use of STAC strategies posttraining (*β*=.34; *P*=.02; 95% CI 0.10-1 .06). In contrast, acceptability was not a significant predictor of posttraining STAC strategy use (*β*=−0.06; *P*=.66; 95% CI −0.35 to 0.23).

## Discussion

### Principal Findings

This is the first study to examine middle school students’ perceptions of the acceptability and relevance of the STAC-T app and the relationship between program acceptability and relevance and posttraining strategy use. The results of this study indicate that the majority of students perceived the program to be both acceptable and relevant. Furthermore, the majority of students who witnessed bullying posttraining also reported use of at least one STAC strategy to intervene when witnessing bullying. Additionally, the results from the regression analysis demonstrated that among students who witnessed bullying posttraining, perceived relevance of the program was a significant predictor of posttraining STAC-T strategy use. In contrast, program acceptability was not significantly related to posttraining strategy use.

### Acceptability and Relevance

Overall, the majority of students perceived the STAC-T program to be both acceptable (188/229, 82.1%, to 206/229, 90.0%) and relevant (180/229, 78.6%, to 190/229, 83%). The findings from this study are consistent with prior research examining the acceptability and relevance of the STAC in-person program for middle school students in low-income communities [17]. Specifically, acceptability ratings in prior research ranged from 83.9% to 88.9%, and relevance ratings ranged from 82.9% to 88.9%. Study results also extend findings from the initial STAC-T app usability testing, which included qualitative data related to acceptability and relevance for the program [26]. The current findings support the acceptability and relevance of the STAC-T app, with acceptability and relevance similar to the in-person STAC intervention, and add to the literature by examining acceptability and relevance among a larger, more diverse sample.

### Use of STAC Strategies

For the use of STAC strategies, among students who reported witnessing bullying posttraining, the majority (111/125, 88.8%) reported using at least one STAC-T strategy. Although slightly lower, this rate is comparable with the in-person STAC intervention research among middle school students, with rates of posttraining STAC strategy use ranging from 91.7% [37] to 95.2% [16]. Furthermore, the results of this study indicated that students were most likely to use *Turning it Over* and *Accompanying Others*, relative to *Stealing the Show* and *Coaching Compassion*. These findings are also consistent with in-person STAC research, demonstrating similar patterns in rates of use by strategy [37]. One possible explanation for the differential strategy use rates is that when using *Stealing the Show* or *Coaching Compassion*, students engage with the student who is bullying either indirectly by attracting their attention or directly by speaking to them. Students may hesitate to engage either indirectly or directly with the student who is bullying, as students may perceive these behaviors to be associated with an increased risk of becoming the target of bullying themselves [38]. Findings suggest that overall, STAC-T posttraining strategy use is similar to rates found for the in-person STAC intervention.

### The Relationship Between Acceptability, Relevance, and Use of STAC Strategies

Finally, the results of this study demonstrated that perceived program relevance, but not acceptability, is uniquely related to use of the STAC strategies. Although bivariate correlations revealed a positive relationship between both program acceptability and relevance, only relevance provided a significant, unique contribution to the variance in use of the STAC strategies. Research indicates that program acceptability is related to outcomes [27], whereas relevance is related to the use of intervention skills [28]. Thus, our finding that program relevance was uniquely related to the posttraining use of the STAC strategies is consistent with prior research. Although intervention success depends on intervention acceptability [27], with acceptability functioning as a gatekeeper for intervention implementation [39], program relevance represents an additional level of social validity. Specifically, program relevance refers to the perception that the intervention is designed for similar users rather than a one-size approach, which can be enhanced by the ability to customize the program [40]. Thus, the ability to select avatars, view the avatar enacting a selected STAC strategy for a specific bullying situation, and receive feedback may enhance program relevance. These findings underscore the importance of program development and program features that promote the perception of program users that the program is relevant to their peer group and students at their school in order for users to apply the skills provided by the training.

### Study Limitations

Although this study adds to the growing body of literature suggesting STAC-T is a promising bullying prevention program for middle school students, there are limitations. First, although participants were recruited from 6 middle schools in 3 geographic regions of the United States to increase generalizability, students from different geographic areas may have different perceptions of acceptability and relevance. Future research with larger, more diverse samples is needed to increase the generalizability of the findings. Additionally, our examination of bystander status and STAC strategy use was limited to self-report data. Retrospective self-report measures of bystander behavior can be biased or inaccurate [41]. Additionally, self-reports of bystander behavior include not just bystander behavior, but the interpretation of the event eliciting the bystander response [42]. Future research could include observational data or teacher-reported data to provide a more robust measure of strategy use. Furthermore, although program relevance was a significant predictor of use of STAC strategies, relevance accounted for only a small amount of variance in strategy use. Thus, unmeasured factors other than program relevance (eg, the actual program content) may account for the majority of variance in the frequency of post-training use of STAC strategies. Furthermore, consistent with the in-person STAC intervention, the findings from this study indicate students are less likely to use Stealing the Show and Coaching Compassion, relative to Turing it Over and Accompanying Others. It is possible that the training examples or avatar activities may need to be modified to be more relevant for middle school students. Qualitative data could provide valuable feedback related to how to encourage students to use Stealing the Show and Coaching Compassion.

### Implications

The results of this study have important implications for the use of technology-based bullying prevention programs for middle school students. First, the findings from this study, coupled with prior research [25,26], indicate middle school students perceive the STAC-T app to be both acceptable and relevant. Furthermore, STAC-T acceptability and relevance rates are consistent with rates for the in-person STAC intervention, suggesting that students perceive the technology version of STAC to be as acceptable and relevant as the in-person intervention. The results of this study also provided support for the effectiveness of the STAC-T app in training students to intervene when they witness bullying using the 4 STAC strategies. Although slightly lower than STAC in-person posttraining skill use rates, STAC-T posttraining rates were comparable. Together, these findings are particularly important as STAC-T requires fewer resources for administration than the in-person STAC intervention and was designed to significantly reduce implementation barriers for schools, particularly those in low-income communities. Thus, this study supports the STAC-T app as a promising technology-based program for bullying prevention among middle school students.

### Conclusions

Bullying is a significant public health issue for middle schools in the United States. Schools face many barriers to accessing and implementing bullying prevention programs. STAC-T is a technology-based bullying prevention app that has the potential to reduce these barriers. Findings from this study demonstrate the program acceptability, relevance, and effectiveness of STAC-T in training students to use skills to intervene when they witness bullying. This study provides support for the acceptability and relevance of the STAC-T app, as well as preliminary evidence for the effectiveness of the program.

## Abbreviations

PIN: personal identification number
VIF: variance inflation factor
